# *Solobacterium moorei* promotes tumor progression via the Integrin α2/β1-PI3K-AKT-mTOR-C-myc signaling pathway in colorectal cancer

**DOI:** 10.7150/ijbs.102742

**Published:** 2025-01-27

**Authors:** Yan Chen, Ying Qin, Tingting Fan, Cheng Qiu, Yijie Zhang, Mengmeng Dai, Yaoyao Zhou, Qinsheng Sun, Yuan Guo, Yue Hao, Yuyang Jiang

**Affiliations:** 1 Guangdong Provincial Key Laboratory of Chinese Medicine Ingredients and Gut Microbiomics, School of Pharmacy, Shenzhen University Medical School, Shenzhen University, Shenzhen, Guangdong, China.; 2 State Key Laboratory of Chemical Oncogenomics, Tsinghua Shenzhen International Graduate School, Shenzhen, Guangdong, China.; 3 Department of Gastrointestinal Surgery, Shenzhen Second People's Hospital, Shenzhen, Guangdong, China.; 4 Institute of Biomedical Health Technology and Engineering, Shenzhen Bay Laboratory, Shenzhen, Guangdong, China.; 5 School of Pharmaceutical Sciences, Tsinghua University, Beijing, China.

**Keywords:** *Solobacterium moorei*, Colorectal cancer, Tumor progression, Pathogen-mediated mechanism, Integrin α2/β1-PI3K-AKT-mTOR-C-myc signaling pathway

## Abstract

More and more evidences show that the imbalance of intestinal flora homeostasis can contribute to the progression of colorectal cancer (CRC).* Solobacterium moorei (S. moorei)*, an anaerobic Gram-positive bacillus, was found to be enriched in fecal samples from CRC patients. However, the signaling regulatory mechanism of *S. moorei* promoting CRC progression remain unknown. Three CRC mouse models (*Apc^Min/+^* mice, AOM/DSS-treated mice and subcutaneous colorectal xenograft mice) and two cell lines (DLD-1 and HT-29) were used to investigate the biological functions and molecular mechanisms of *S. moorei* on tumor progression of CRC *in vivo* and *in vitro*. *S. moorei* abundance increased in fecal samples and tumor tissues, and was significantly positively correlated with tumor staging of CRC. *S. moorei* promoted tumor progression in various CRC mouse models and it selectively adhered to cancer cells in comparison to colonic mucosal epithelial cells, enhancing CRC cell proliferation and inhibiting cell apoptosis. Mechanistically, *S. moorei* cellwall protein Cna B-type domain-containing protein binds to integrin α2/β1 on CRC cells, leading to the activation of PI3K-AKT-mTOR-C-myc pathway via phospho-FAK, thereby promoted tumor cell growth and progression. Blockade of integrin α2/β1 abolished *S. moorei*-mediated oncogenic response *in vitro* and *in vivo*. In summary, this study demonstrated that *S. moorei* promoted tumor progression via the integrin α2/β1-PI3K-AKT-mTOR-C-myc signaling pathway, which is a novel specific pathogen-mediated mechanism that might be a new potential target for CRC prevention, diagnosis, and treatment.

## Introduction

Maintaining intestinal flora homeostasis is crucial for human health, and the imbalance of intestinal flora homeostasis leads to disease conditions^1^, ^2^. An increasing number of research studies have shown a strong link between intestinal flora and the progression and treatment of colorectal cancer (CRC) in humans^3^-^5^. Recently, the oral microbiome is recognized as a critical factor that affects intestinal flora homeostasis^6^. Oral microbiota is the second largest human microbiota after intestinal microbiota, and most oral microbial strains are delivered from the oral cavity to the large intestine^7^. Oral microbes have been increasingly recognized to be tightly associated with CRC^8^, ^9^. So far, oral bacteria such as* Fusobacterium nucleatum*, *Porphyromonas gingivalis*, *Parvimonas micra* and *Peptostreptococcus anaerobius*, were shown to invade human intestinal epithelium, which promoted the development of colorectal adenoma by several possible mechanisms^10^-^12^.

*Solobacterium moorei* (*S. moorei*) is a gram-positive, non-spore-forming, anaerobic bacillus^13^. Unlike* Fusobacterium nucleatum, Porphyromonas gingivalis* and so on, *S. moorei* is an oral pathogen that has not been widely concerned. *S. moorei* was first identified in human fecal samples and described as a member of the oral microbiota^14^. It is a unique member of the genus *Solobacterium* in Clostridium cluster XVI. *S. moorei* should also be considered as an opportunistic pathogen, responsible for severe infections. *S. moorei* has been implicated in bacteraemia, necrobacillosis-associated thrombophlebitis, septicemia, and wound infection^15^, ^16^. *S. moorei* also has been detected in endodontic infections, periradicular lesions, and subgingival plaque of refractory periodontitis^17^, ^18^. In recent years, it has been found to be associated with oral malodour^19^, ^20^ and oral squamous cell carcinoma^21^. Several microbiome sequencing studies reported that *S. moorei* is selectively enriched in the mucosal microbiota and fecal samples from CRC patients^22^-^24^.

*S. moorei* has been reported to promote the progression of colon adenomas by causing inflammation and disrupting the intestinal barrier, demonstrating its important role in the process of inflammation-cancer transformation^25^. However, the relevant signal regulatory mechanisms are unknown. In addition, studies have shown that *S. moorei* exhibited a stronger replication rate during the adenoma phase, while its abundance significantly increased during tumor progression^23^, indicating that in addition to playing a role in the early occurrence of CRC, *S. moorei* may also promote tumor development by regulating the proliferation of CRC cells. This suggests that *S. moorei* may become a potential candidate target for prevention and treatment of CRC. Therefore, in-depth research on the signaling pathways and key molecule mechanism involved in the regulation of tumor cell proliferation by *S. moorei* has great scientific and clinical value.

This study investigated the abundance of *S. moorei* in the feces and tumor of CRC patients and assessed its role in CRC development using mouse models and CRC cell lines. We found that *S. moorei* enhanced CRC occurrence by upregulating integrin α2/β1 expression in CRC cells, which is known to be overexpressed in human CRC. The interaction between integrin α2/β1 and *S. moorei* cellwall protein Cna B-type domain-containing protein activated the PI3K-Akt-mTOR pathway, resulting in C-myc activation and promoting tumor growth and progression. Our findings reveal a novel pathogen-mediated mechanism contributing to CRC progression.

## Materials and methods

### Analysis based on GMrepo database

GMrepo database was used to obtain data on microbial associations to host phenotypes for different species^26^. GMrepo (data repository for Gut Microbiota) is a database of curated and consistently annotated human gut metagenomes. The abundances of *S. moorei* in fecal samples of patients with CRC and healthy subjects were obtained from GMrepo and the relative abundance of *S. moorei* was analyzed. More detailed information is available on gmrepo.humangut.info/help.

### Patients and clinical specimens

Clinical fecal specimens were respectively obtained from 96 healthy subjects and 89 CRC patients diagnosed by colonoscopy and biopsy from the health examination center and department of gastrointestinal surgery of Shenzhen Second People's Hospital. For healthy subjects, diarrhea was not reported in the past one month. Use of antibiotics and probiotics was not permitted from 1 month before the specimen collection. Freshly collected fecal specimens from CRC patients and healthy specimenwere frozen immediately after collection and preserved at -80 °C. The primary CRC tissues and the corresponding non-tumor tissues (5 cm away from the tumor edge) were obtained from patients who underwent surgery and were collected in RNAlater™ Stabilization Solution (Thermo Fisher Scientific, Waltham, MA, USA) and preserved at -80 °C refrigerator before analysis. The clinicopathological features of the samples are displayed in Table S1. CRC patients included in this study did not receive any treatment before surgery.

### DNA and RNA extraction and qRT-PCR

QIAGEN stool kit (QIAGEN, Germany) was used to extract bacterial DNA from the fecal specimens of humans and mice, and QIAGEN DNA mini kit (QIAGEN, Germany) was used for extracting bacterial genomic DNA (gDNA) from human and mouse colonic samples. TRIzol reagent (Thermo Fisher Scientific) was used to isolate total RNA from cells and tissues. cDNA was synthesized by BeyoRT™ II thesis kit. RNA (1µg) was used for the reverse transcription reaction. Genomic DNA contamination was eliminated with gDNA Eraser kit (Beyotime Bio Inc, China). Thereafter, qRT-PCR assay was performed using a 7500 PCR system (Thermo Fisher Scientific). The PCR reaction conditions were as follows: initial denaturation at 95 °C for 5 min, followed by 40 cycles of denaturation at 95 °C for 10s, annealing and extension at 60 °C for 35s (Takara Bio, Japan). The relative abundance of *S. moorei* was calculated by the comparative CT method (2^-ΔCt^ method in fecal specimens and 2^-ΔΔCt^ method in tissue specimens). Universal eubacteria 16s rDNA was used as endogenous control. Further, the relative expression of the mRNA of the target genes was calculated by the 2^-△Ct^ method, with GAPDH serving as a positive control. Primer sequences are enlisted in Table S2^11^, ^12^, ^27^-^31^.

### Bacterial Culture

*S. moorei* strain JCM10645 was purchased from the Japan Collection of Microorganisms (JCM, Japan). The hypervariable regions (V1-V9) of 16S ribosomal RNA (rRNA) were sequenced to confirm the identity of bacterial species. EG Broth Medium (BD Difco, Sparks, MD, USA) was used to grow bacteria in an anaerobic jar (80% N_2_, 10% CO_2_, and 10% H_2_) at 37 °C. Thereafter, cultures were collected, centrifuged at 3000 rpm for 5 min at 4 °C, washed twice with sterile anaerobic PBS, and resuspended at a concentration of 1 × 10^8^ colony-forming units (CFU)/200 µL in an anaerobic environment for subsequent animal experiments. Meanwhile, *E. coli* strain MG1655 (ATCC 47076), a non-pathogenic human commensal gut bacterium, was used as a negative control (NC) for *S. moorei* infection. *E. coli* strain MG1655 was grown in lysogeny broth (LB) under aerobic conditions.

### Colorectal adenoma mouse model

6-week-old C57BL/6 *Apc^Min/+^* male mice (GemPharmatech, Jiangsu, China) and C57BL/6 wild type male mice (Medical laboratory animal center, Guangdong, China) were administered drinking water containing antibiotics (0.1g/L vancomycin, 0.2 g/L ampicillin, metronidazole, and neomycin) for 2 weeks. For the *Apc^Min/+^* model, animals were randomized into three groups at one-week post-treatment, including mice receiving oral gavage of *S. moorei* (1 × 10^8^ CFU), *E. coli* MG1655 (1 × 10^8^ CFU), and PBS once every two days for 10 weeks. For the AOM/DSS-treated model, animals were randomized into four groups, (A) Normal control group: normal control with gavage daily PBS; (B) Model control group: AOM/DSS with gavage daily PBS; (C) *E. coli* group: AOM/DSS plus gavage 1 × 10^8^ CFU *E. coli* suspension; (D) *S. moorei* group: AOM/DSS plus gavage 1 × 10^8^ CFU *S. moorei* suspension. NC group received an injection of sterile saline, whereas the rest of the groups received an intraperitoneal injection of azoxymethane (AOM, Sigma-Aldrich, USA) solution at a doge of 10 mg/kg body weight at the beginning of the experiment. NC group was administered drinking water, whereas other groups were administered drinking water containing 2.5% dextran sulfate sodium salt (DSS, Yeasen, China) for 5 days followed by 14 days of normal water, this process repeated for three cycles. The bacteria were applied by oral gavage once every two days except when DSS was applied. After the completion of the experiment, mice were sacrificed, and the number and the volume of tumors or colon length in colon cancer mice were determined. Colon cancer tissues and matched non-tumor tissues were harvested, snap-frozen in liquid nitrogen, and preserved in 10% buffered formalin before use.

### Subcutaneous colorectal xenograft mouse model

For the integrin α2/β1 blockade, DLD-1 cells (2 × 10^6^) were inoculated into the left armpit of BALB/c-nu mice at 5 weeks of age. When the tumor grew to 100 mm^3^, the mice were randomly divided into four groups, receiving multipoint intratumoral injection of EG medium, *S. moorei* (1 × 10^8^ CFU), RGD peptides (100 µg), and *S. moorei* plus RGD peptides once every two days for 2 weeks. The mice were euthanized before the tumors were dissected.

### Histopathological analysis

Formalin-fixed paraffin-embedded tissues were used for histopathological analysis. 5 mm sections were prepared, stained with Haemotoxylin and Eosin (H&E), and used for histopathological analysis. H&E-stained sections were evaluated by experienced pathologists blinded to the treatments.

### Cell Culture

Colon cancer cell lines DLD-1 and HT-29 were acquired from Cell Bank, China Academy of Sciences (Shanghai, China). Immortalized human colonic epithelial cell NCM460 was kindly provided by BeNa Culture Collection (Henan, China). DLD-1, HT-29, and NCM460 cells were cultured in RPMI-1640, McCoy's 5A, and DMEM (Gibco, Thermo Fisher Scientific), respectively. Each media contained 10% (v/v) fetal bovine serum (FBS), and the cells were maintained in a humidified incubator at 37 °C with 5% CO_2_. Each cell line was recently authenticated by STR profiling and tested for mycoplasma contamination. For the bacterial infection study, cells were infected with *S. moorei* anaerobically or *E. coli* MG1655 at MOI (multiplicity of infection) of 100 for 2 h every 24 hours. Following incubation, then removed the bacteria and the bacteria-containing medium was substituted by a cell culture medium that contained 10% FBS, 20 µg/mL gentamycin, and 1% penicillin-streptomycin (PS) continue to culture cells in the carbon dioxide incubator. Cells were co-cultured with bacteria three times within 72 hours.

### Bacterial attachment assay

To study the attachment of *S. moorei* on colonic cells, NCM460, DLD-1, and HT-29 cells were co-cultured with 100 MOI for 2h in anaerobically condition, at that time *S. moorei* attached to cell surface(infected). After wash with PBS for 3 times, *S. moorei* which did not attach would be wash off. Then 10,000 cells attached with bacteria were plated in EG solid medium media which only allowed *S. moorei* to grow. Finally, we counted the formation of colony of *S. moorei* which represent the number of bacteria attached to cell surface. *E. coli* strain MG1655 was used as a negative control (NC). Each cell experiment was conducted thrice.

### Transmission Electron Microscopy

Approximately 1 × 10^6^ CRC cells were grown in a 6-well plate and co-cultured with *S. moorei* for 2h (MOI=10), then fixed in 2.5% glutaraldehyde. Fixed cell samples were further fixed using 1% osmium tetroxide, followed by dehydration with an increasing concentration gradient of ethanol and acetone. Samples were then embedded as followed: acetone: EMBed 812 epoxy = 1:1 for 2-4 h at 37°C; acetone: EMBed 812 epoxy = 1:2 overnight at 37°C; pure EMBed 812 epoxy for 5-8 h at 37°C. The resin blocks were cut to 60-80nm thin on the ultra-microtome, and the samples were fished out onto the 150 meshes cuprum grids with formvar film, then stained with 2% uranium acetate saturated alcohol solution and 2.6% lead citrate. The cuprum grids are observed under HITACHI TEM system and take images.

### Cell Proliferation Assay

Nearly 5000 CRC cells were plated into a 96-well plate and co-cultured with *S. moorei* or *E. coli* MG1655 (MOI = 100) for 2 h every 24 hours, then removed the bacteria and continue to culture cells in the carbon dioxide incubator. CCK8 (Yeasen, China) kit was utilized to measure CRC cell viability in 24, 48, 72 hours after inoculation with *S. moorei* as described in the manufacturer's protocol. For the colony-forming assay, 1000 CRC cells were plated into a 24-well plate and also incubated with *S. moorei* or *E. coli* MG1655 (MOI = 100) for 2 h every 24 hours. After 72 hours, 1000 CRC cells were inoculated in a 6-well plate and cultured for 10 days. Following incubation, viable colonies (>50 cells/colony) were counted and stained with Giemsa. Each cell experiment was conducted thrice.

### Cell apoptosis analysis

The cell apoptosis analysis was performed using flow cytometry. Cells were seeded into six-well plates and incubated with *S. moorei* or *E. coli* MG1655 (MOI = 100) for 2 h every 24 hours. After 72 hours, according to the manufacturer's instructions, apoptotic cells were measured with FITC AnnexinV Apoptosis Detection Kit (Yeasen, China).

### RNA-sequencing analysis

Gene expression profiles of DLD-1 and HT-29 cells treated with or without *S. moorei* were analyzed by RNA sequencing. Nearly 1 × 10^6^ colonic cells were infected with bacteria (MOI = 100) in a 6-well plate and maintained anaerobically for 4 hours. Cells were rinsed with PBS, and TRIzol reagent (Thermo Fisher Scientific) was utilized to extract total cellular RNA. RNA-seq was performed by BGI Technology Inc (Shenzhen, China). Gene Ontology (GO) analysis was performed to identify significantly enriched pathways using Metascape database^32^ with the following criteria: the threshold of minimal count = 3, p-value < 0.01, and enrichment factor > 1.5.

### Western blotting

Total cellular proteins were separated through SDS-PAGE and transferred on a PVDF membrane (BioRad, Hercules, CA). The membrane was incubated with primary and secondary antibodies sequentially (Table S3). The protein-antibody complex was detected using an enhanced chemiluminescence (ECL) detection system. Image analysis was performed to quantify protein expression levels with GAPDH as an internal control. Representative images of three independent experiments are shown in the Figures.

### Knockdown of integrin α2/β1 within CRC cells

Lipofectamine 3000 Reagent (Invitrogen) was used for transfecting cells with small interfering RNAs (siRNA) specific for ITGA2 and ITGB1 (GenePharma, China) according to the manufacturer's protocol. Scrambled siRNA was used as a control. siRNA sequences utilized for inhibiting the expression ITGA2 and ITGB1 are displayed in Table S4. Cells transfected with siRNAs for 24 h were harvested for subsequent experiments.

### Mass spectrometry identification of *S. moorei* proteins

1ⅹ10^9^ amount of *S. moorei* were lysis and the protein were extracted through Gram-Positive Bacterium Protein Extraction Kit (Phygene, China). SDS-PAGE were performed and the protein gel were obtained for further LC/MS. The identification of protein gel strips is to separate the sample proteins by gel electrophoresis, then obtain the protein gel strips at different positions on the film, extract the peptides after enzymatic digestion. Separation was performed by Easy-nLC 1200 (Thermo Fisher Scientific, San Jose, CA). The nanoliter liquid phase separation end was directly connected to the mass spectrometer. The separated peptides were then ionized using a nanoESI source and analyzed by the Orbitrap Exploris 480 mass spectrometer (Thermo Fisher Scientific) in DDA mode. At last, the protein identification software which refer to Uniport database was used to identify the proteins in the samples.

### Far-western assay

Whole-cell *S. moorei* proteins were extracted with Gram-Positive Bacterium Protein Extraction Kit (PH1710, Phygene), separated through SDS-PAGE and transferred on a PVDF membrane. The PVDF membrane was blocked with 5% BSA for 2h and incubated with human integrin α2/β1 heterodimer protein (ACRO Biosystems) overnight at 4 °C. The membrane was incubated with integrin α2/β1 and IgG primary antibody respectively for 12h at 4°C. After wash with TBST, membrane incubated with secondary antibody sequentially. The protein-antibody complex was detected using an ECL detection system.

### Molecular docking for Integrinα2/β1 and Cna B-type domain-containing protein

Rigid protein-protein docking was performed between Integrinα2/β1 and Cna B-type domain-containing protein to investigate the relationships by using GRAMM-X (http://gramm.compbio.ku.edu/). The predicted protein structural domains of Integrinα2/β1 and Cna B-type domain-containing protein were obtained from the Alpha Fold 2 (https://alphafold.com/) and ITASSER (https://zhanggroup.org/I-TASSER/). Pymol (Version 2.4) and PDBePISA (https://www.ebi.ac.uk/pdbe/pisa/) were used to investigate protein-protein interactions and further visual analysis.

### Expression and purification of Cna B-type domain-containing protein

Expression of Cna B-type domain-containing protein with His-tag was carried out in *E. coli* BL21(DE3) cells transformed with plasmids. Recombinant BL21(DE3) stored in glycerol was inoculated into TB medium containing related antibiotic and cultured at 37°C. When the OD600 reached about 1.2, cell culture was induced with 0.5mM IPTG at 15°C for 16h and was harvested by centrifugation. Recombinant BL21(DE3) were resuspended with lysis buffer followed by sonication. The precipitate after centrifugation was dissolved using denaturing agent. Target protein was obtained by two-step purification using Ni column+Q Sepharose column. Target protein after refolding was sterilized by 0.22 μm filter before stored in aliquots. The concentration was determined by Bradford protein assay with BSA as standard. The protein purity and molecular weight were determined by standard SDS-PAGE confirmation. Next, DLD-1 and HT-29 cells were treated with recombinant Cna B-type domain-containing protein at concentrations of 0, 0.05, 0.2, and 1 μg/ml, and the effects of the Cna B-type domain-containing protein on the proliferation and apoptosis of CRC cells were verified through CCK8 assay, colony-forming assay and flow cytometry.

### Co-Immunoprecipitation

To validate interaction of Cna B-type domain-containing protein and integrin α2/β1, co-immunoprecipitation assay was performed. Briefly, 1ⅹ10^7^ DLD-1 cells and 1ⅹ10^9^ *E. coli* BL21(DE3) cells expressing Cna B-type domain-containing protein with His-tag were lysis and protein were extracted. Mixture of 200μL DLD-1 protein and *E. coli* protein were added and coupled to 50μL IgG Magnetic Beads (Beyotime, China) and Anti-His Magnetic Beads (Beyontime, China) for 24h, respectively. The beads are washed to remove unbound proteins, and the bound proteins are eluted with elution buffer. The eluted proteins are analyzed by Western blotting using specific antibodies to detect Cna B-type domain-containing protein with His-tag, integrin α2 and β1.

### Statistical analysis

Statistical significance between the two groups was calculated by the student's t-test or Mann-Whitney U-test. One-way ANOVA was adopted to assess the statistical significance of cell growth curves. All *in vitro* experiments were repeated at least three times, results were represented by mean ± s.d. P < 0.05 (two-tailed) indicated statistical significance. *p < 0.05, **p < 0.01, ***p < 0.001, ****p < 0.0001, ns: no significant.

## Results

### *Solobacterium moorei* enrichment in fecal and tumor specimens from CRC cases

**Figure 1:**
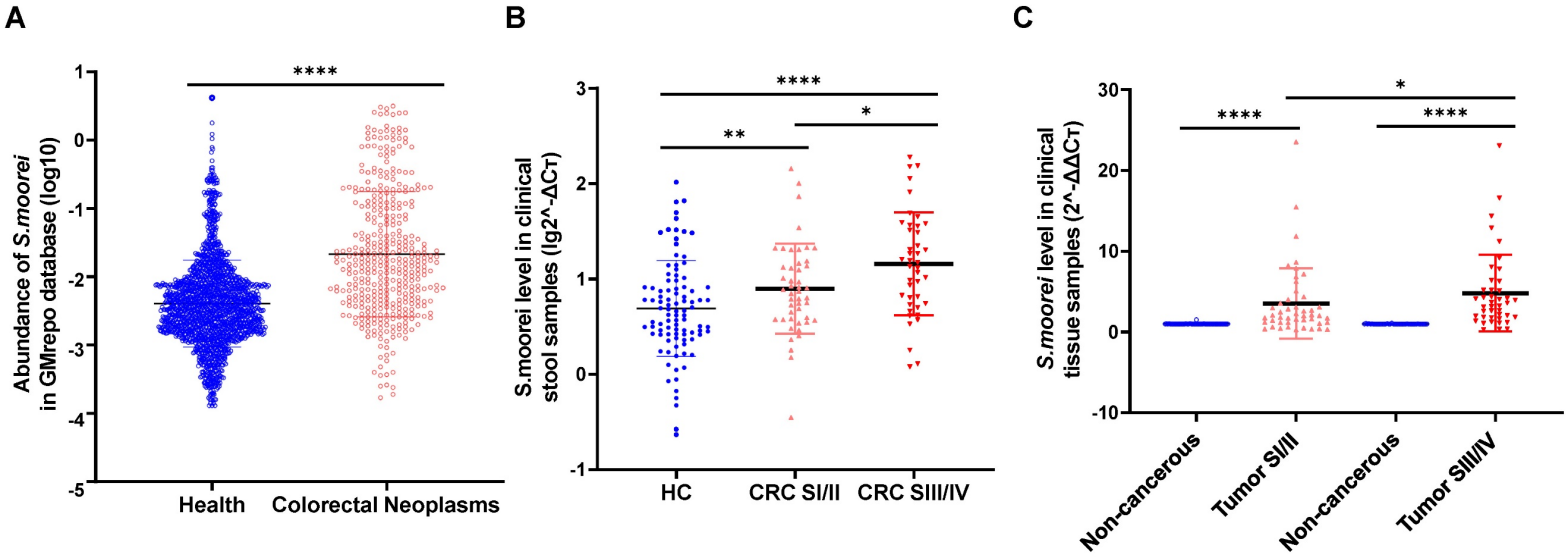
** The clinical relevance of *S. moorei* abundance with CRC.** (A) The fecal abundance of *S. moorei* in the healthy controls (n =1589) and CRC patients (n =390) in GMrepo database. (B) The level of *S. moorei* in stool samples from healthy controls (n = 96), stage I/II CRC patients (n = 47) and stage III/IV CRC patients (n = 42). (C) The level of *S. moorei* in the tumor and non-carcinoma tissues from CRC patients (stage I/II, n = 47; stage III/IV, n = 42). HC, healthy control; CRC, colorectal cancer; SI/II, stage I/II; SIII/IV, stage III/IV.

We first analyzed *S. moorei* levels in the fecal samples of CRC cases and healthy subjects from GMrepo database. *S. moorei* levels were higher in CRC cases (n=390) in comparison to the healthy control samples (n=1589) (Figure 1A). To understand the association of *S. moorei* with CRC, the levels of *S. moorei* in the fecal specimens of 89 CRC cases and 96 healthy subjects were examined by qRT-PCR. *S. moorei* abundance was remarkably elevated in fecal specimens from CRC cases relative to healthy subjects and a significant association was observed between the *S. moorei* abundance in stools with the CRC tumor staging (Figure 1B). The abundance of *S. moorei* in the feces of stage III/IV CRC patients was significantly higher than that of stage I/II CRC patients. The abundance of *S. moorei* was also examined in tumor tissues and matched non-tumor tissues from CRC patients (Figure 1C). *S. moorei* abundance was remarkably elevated in tumor tissues and was also significantly correlated with tumor staging, suggesting the possible involvement of *S. moorei* in the progression of CRC.

### *S. moorei* promotesd CRC occurrence among *Apc^Min/+^* and AOM/DSS-treated C57BL/6 Mice

**Figure 2:**
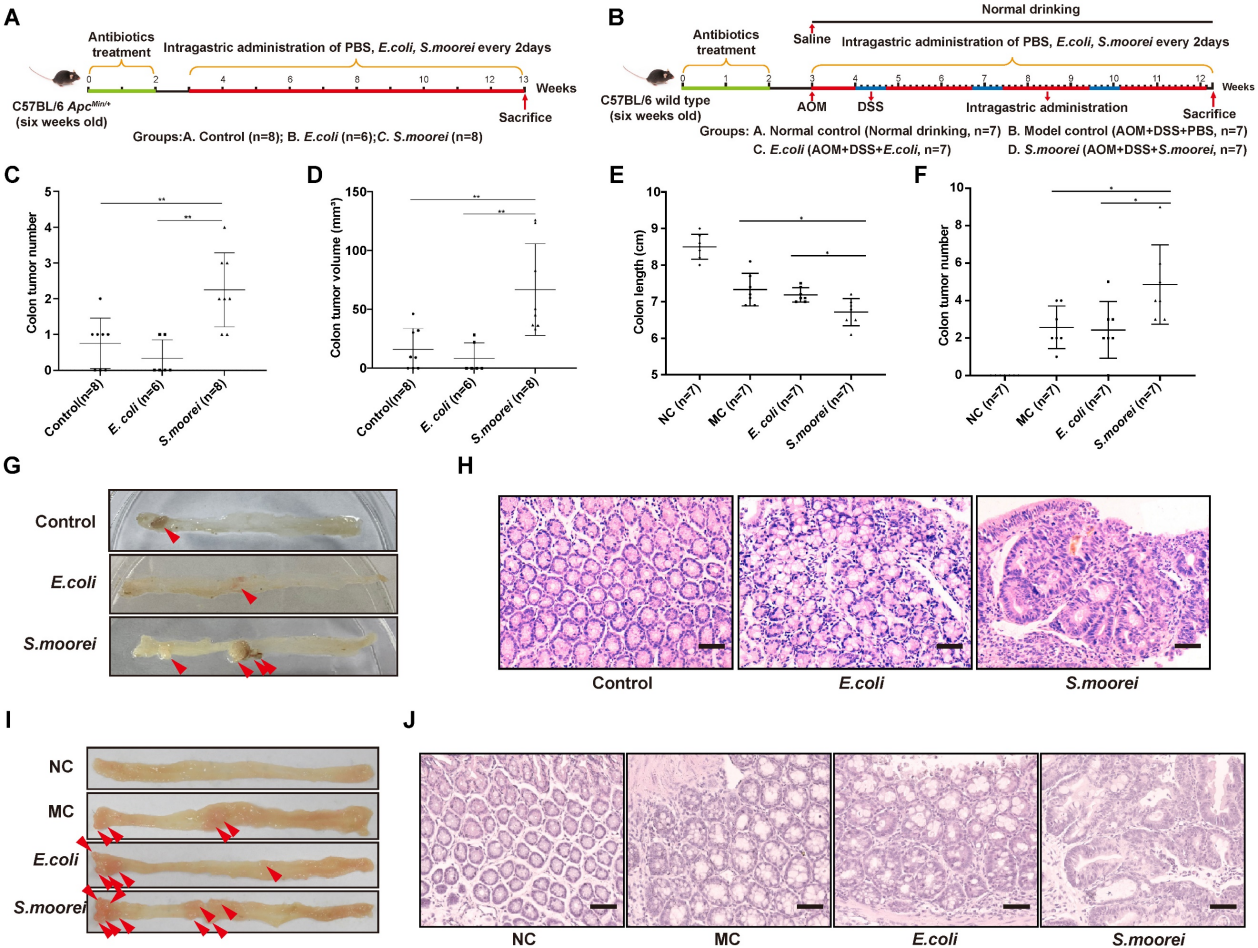
***S. moorei* promotes colonic tumorigenesis in *Apc^Min/+^* and AOM/DSS-treated mice.** (A) Schematic diagram showing the experimental design and timeline of *Apc^Min/+^* mouse models. (B) Schematic diagram showing the experimental design and timeline of AOM/DSS-treated mouse models. (C) The colonic tumor number of *Apc^Min/+^* mice under different treatments. (D) The tumor volume of *Apc^Min/+^* mice under different treatment. (E) The colon length of AOM/DSS-treated mice under different treatment. NC, normal control; MC, model control. (F) The colonic tumor number of AOM/DSS-treated mice under different treatments. (G) Representative colonic morphologies of *Apc^Min/+^* mice under different treatment. (H) Representative histological images of colon tissues of *Apc^Min/+^* mice by H&E staining. Scale bars, 50 µm. (I) Representative colonic morphologies of AOM/DSS-treated mice under different treatment. NC, normal control; MC, model control. (J) Representative histological images of colon tissues of AOM/DSS-treated mice by H&E staining. Scale bars, 50 µm.

To assess the effect of *S. moorei* on CRC occurrence, the Spontaneous tumorigenesis model (*Apc^Min/+^*) and inducing tumorigenic model (AOM/DSS-treated) were utilized (Figure 2A-B). To deplete microbiota, antibiotics were administered to all mice for two weeks before the treatment. 1 × 10^8^ colony-forming units (CFUs) of *S. moorei* were administered to each mouse via oral gavage in every 2 days in both *Apc^Min/+^* and AOM+DSS models until the end of experiment. In addition, PBS or *E. coli* strain MG1655 (1 × 10^8^ CFU) was administered to the mice as reference. qRT-PCR analysis of *S. moorei* in feces during feeding period confirmed that *S. moorei* colonized the intestinal tract of mice after inoculation (Figure S1A). The tumor development was examined at the end of experiment. Mice treated with *S. moorei* had markedly increased tumor multiplicity and tumor volume relative to those exposed to PBS or *E. coli* treatment (Figure 2C-F). Representative images of the colon from different treatment groups are displayed in Figure 2G-H. All colonic tumors were confirmed histologically, *S. moorei* strongly induced dysplasia in murine colon compared with those treated with *E. coli* MG1655 or PBS in both *Apc^Min/+^* mice and AOM+DSS model (Figure 2I-J). qRT-PCR assay revealed an elevated level of *S. moorei* in colonic tumors compared to matched non-tumor samples (Figure S1B). Our data indicated that *S. moorei* accelerated colorectal tumorigenesis in both *Apc^Min/+^* and AOM+DSS mice.

### *S. moorei* attached preferentially to CRC cells

**Figure 3:**
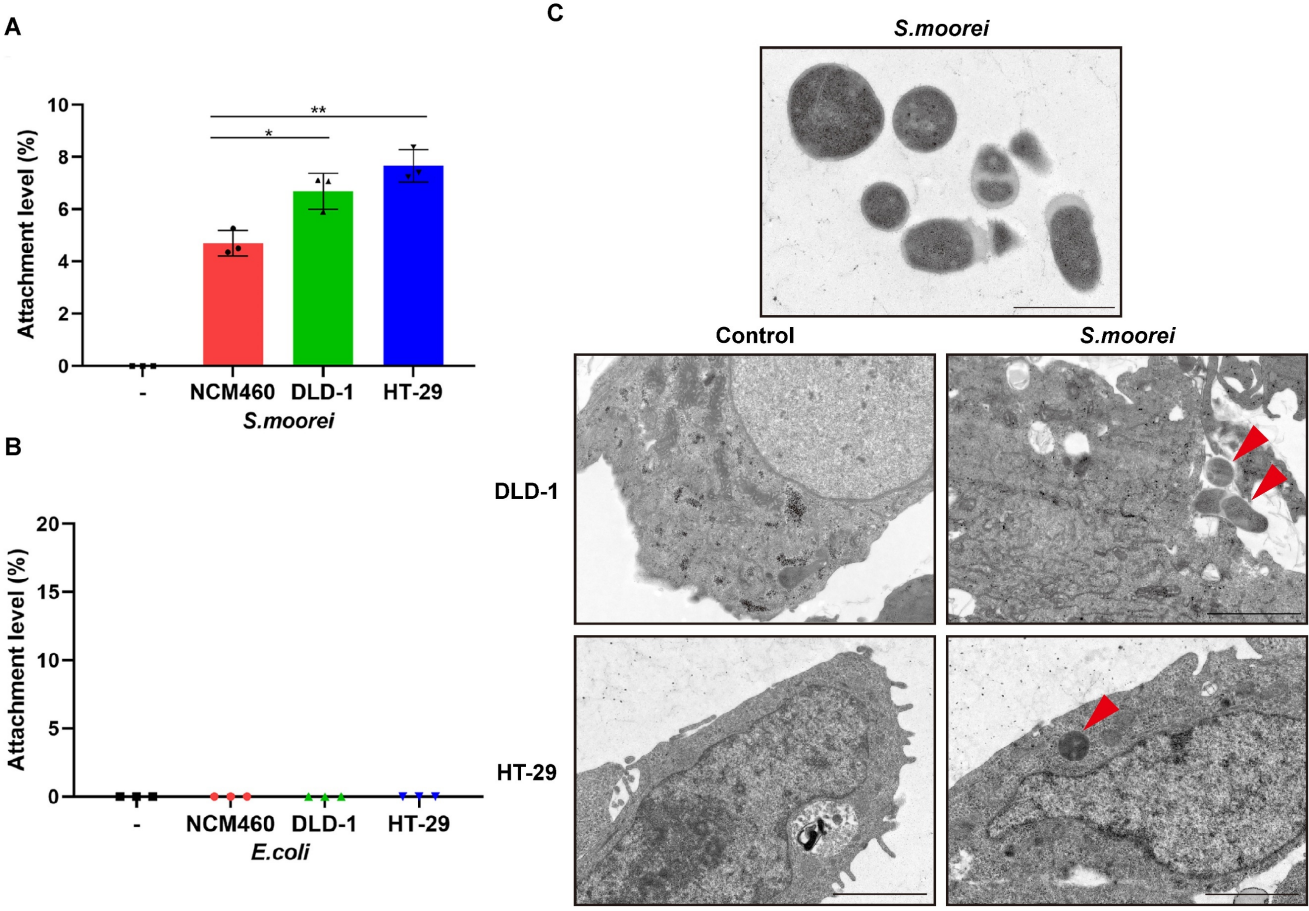
***S. moorei* attaches preferentially to CRC cells.** (A) The level of attachment of *S. moorei* on colon cancer cell lines DLD-1 and HT-29 and the normal colon cell line NCM460. (B) The level of attachment of *E. coli* (MG1655) on colonic cells. (C) Representative TEM images of *S. moorei* attaching to colon cancer DLD-1 and HT-29 cells. The red arrows indicate *S. moorei*. Scale bars, 1 μm in *S. moorei* (The uppermost panel); 2 μm for the remaining panels.

*S. moorei* were known to colonize in colonic tumors of *Apc^Min/+^* mice; therefore, we analyzed *in vitro* adhesion characteristics of *S. moorei* to CRC cells (DLD-1 and HT-29) as well as human colonic mucosal epithelial cells (NCM460). We found that *S. moorei* successfully adhered to all the 3 cell types; however, the attachment efficiency of *S. moorei* was markedly elevated for CRC cells compared to primary epithelial cells (Figure 3A). On the contrary, *E. coli* strain MG1655 showed no obvious attachment to the three cell types (Figure 3B). Consequently, *S. moorei* possibly exhibited oncogenic activity by directly binding to colonic epithelial cells, especially CRC cells. Further, we also performed transmission electron microscopy (TEM) to confirm that *S. moorei* attaches to CRC cells (Figure 3C).

### *S. moorei* induced cell proliferation *in vitro*

**Figure 4:**
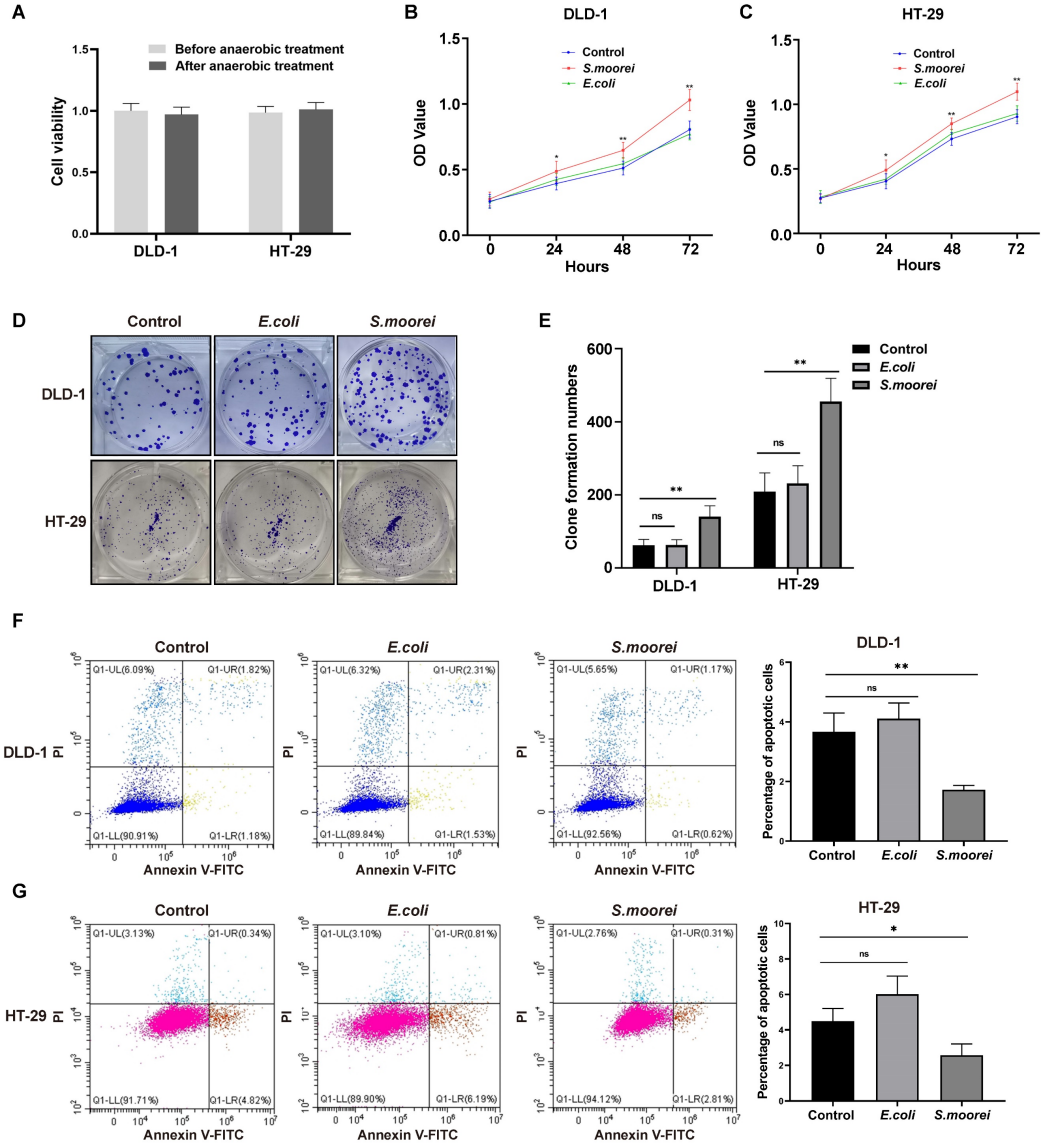
***S. moorei* promotes cell proliferation and inhibits cell apoptosis in CRC cells.** (A) Viabilities of CRC cells under anaerobic incubation condition for 2 hours were not altered. (B) Co-cultured with *S. moorei* promoted cell proliferation of DLD-1 but *E. coli* has no effect on cell viability. (C) *S. moorei* promoted cell proliferation of HT-29 cells. (D) Co-cultured with *S. moorei* promoted colony formation of DLD-1 and HT-29 cells but *E. coli* has no effect. (E) The colony formation number of DLD-1 and HT-29 treated with *S. moorei*. (F) Co-cultured with *S. moorei* inhibited cell apoptosis of DLD-1 but *E. coli* has no effect. (G) *S. moorei* inhibited cell apoptosis of HT-29 cells.

To further investigate the tumorigenic ability of *S. moorei*, the effect of *S. moorei* on cell proliferation was examined *in vitro*. Anaerobic culture of CRC cells for 2 hours had no effect on cell viability (Figure 4A). Additionally, *S. moorei* markedly enhanced the proliferation and the clone forming of DLD-1 and HT-29 cells, but the proliferation of CRC cells did not alter when co-cultured with the control bacteria* E. coli* (Figure 4B-E). Furthermore, Flow cytometry showed that *S. moorei* could inhibit the apoptosis of DLD-1 and HT-29 cells, while *E. coli* had no significant effect on cell apoptosis (Figure 4F-G). We also analyzed the expression of proteins involved in cell proliferation, cell cycle, and apoptosis in CRC cells following treatment with *S. moorei*, *S. moorei* increased the expression of PCNA, Cyclin D1, Bcl-2 and reduced Bax expression (Figure S2A-B). Taken together, our data suggested that *S. moorei* induced the tumorigenicity of CRC by accelerating cell proliferation and inhibiting the cell apoptosis.

### *S. moorei* promoted colorectal carcinogenesis by activating the integrin α2/β1-PI3K-AKT-mTOR-c-Myc signaling pathway

**Figure 5:**
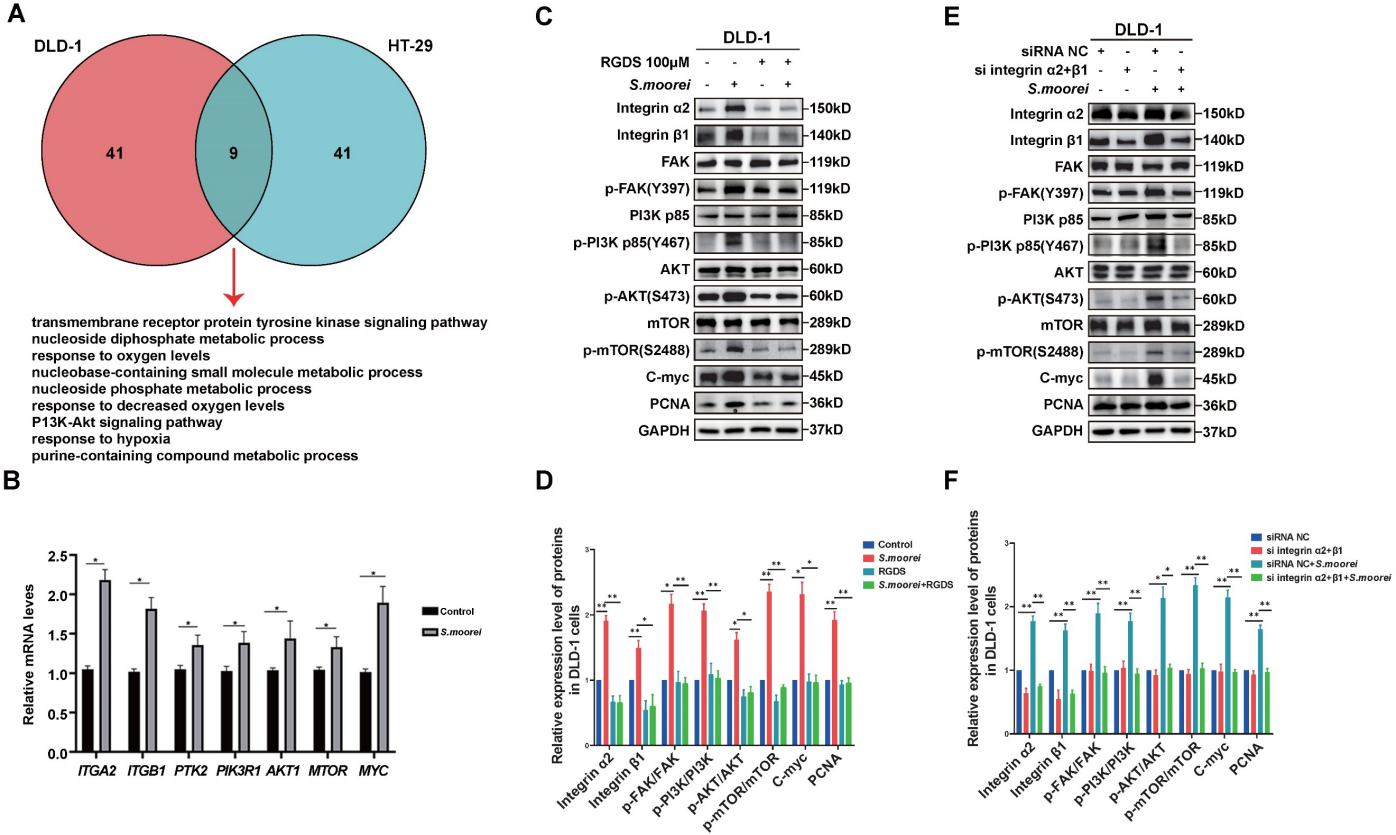
***S. moorei* mediates integrin α2/β1 signaling to activate the FAK-PI3K-AKT-mTOR-C-myc pathway to promote cell proliferation *in vitro*.** (A) *S. moorei* regulated genes enrichment in numbers of pathways in DLD-1 and HT-29 cells. Nine pathways were commonly enriched in the two CRC cell lines. (B) Gene expression of integrin α2/β1-FAK-PI3K-AKT-mTOR-C-myc signaling pathway in DLD-1 cells co-cultured with *S. moorei*. (C) Protein expression of integrin α2/β1-FAK-PI3K-AKT-mTOR-C-myc signaling pathway triggered by *S. moorei* in DLD-1 cells. The effects of *S. moorei* were abolished by the integrin inhibitor RGD peptides (100 µM). RGDS, RGD peptides. (D) Quantification of protein expression for all blots showed in (C). (E) Integrin α2/β1 knockdown abolished the effect of *S. moorei* on the integrin α2/β1-FAK-PI3K-AKT-mTOR-C-myc pathway in DLD-1 cells. (F) Quantification of protein expression for all blots showed in (E).

To understand the mechanism of *S. moorei*-induced CRC occurrence, gene expression levels in DLD-1 and HT-29 cells co-cultured with *S. moorei* were analyzed (Tables S5 and S6). To identify key cellular pathways activated after bacterial treatment, we utilized the Metascape database for GO as well as KEGG analysis. The enriched GO terms and KEGG pathways in DLD-1 and HT-29 cells following co-culture with *S. moorei* were identified (Tables S7 and S8). Based on the 50 most significantly enriched functions and pathways with p < 0.01, a Venn diagram of the significantly altered pathways common in two CRC cell lines was constructed. Overall, 9 significantly altered pathways in two CRC cell lines were identified, including the transmembrane receptor protein tyrosine kinase pathway, metabolism of nucleotides, response to oxygen level variations, and PI3K-Akt signaling pathway (Figure 5A). Integrins play an important role in apical junction remodeling by inducing the phosphorylation of focal adhesion kinase (FAK), which subsequently phosphorylate the p85 subunit of PI3K and activate the PI3K-AKT pathway^33^, ^34^. From the RNA-seq analysis, 356 genes (p < 0.05) were upregulated in CRC cell lines co-cultured with *S. moorei* (Table S9). Among those genes, ITGA2 and ITGB1 are the integrin family genes which activate the PI3K-AKT pathway in *Peptostreptococcus anaerobius*-mediated oncogenic response in CRC^12^. Meanwhile, MYC which is the downstream gene of PI3K-AKT pathway is also up-regulated. Therefore, we assumed that integrin α2/β1-PI3K-AKT-C-myc signaling pathway might be responsible for the tumor-promoting activity of *S. moorei*. For further validation, we analyzed the expression levels of genes involved in the PI3K-AKT pathway. We found that key genes related to the PI3K-AKT pathway were significantly increased in CRC cell lines co-cultured with *S. moorei*, including ITGA2, ITGB1, PTK2(FAK), PIK3R1, AKT1, MTOR and MYC (Figure 5B and S3A). Moreover, *S. moorei* induced the expression of several proteins, including Integrin α2/β1, p-FAK, p-PI3K (p-p85), p-AKT, p-mTOR and C-myc (Figure 5C-D and S3B-C). Our findings suggested that *S. moorei* promoted Integrin α2/β1-PI3K-AKT-mTOR-C-myc signaling pathway in CRC cells.

### Blockade of integrin α2/β1 inhibited tumorigenic and signaling activities of *S. moorei* in CRC cells

To investigate the role of integrin α2/β1 signaling in *S. moorei*-mediated tumorigenic activity of CRC cells, the function of integrin α2/β1 was suppressed by integrin inhibitor RGD peptides or integrin α2/β1 small interfering RNA. Co-culture of CRC cells with *S. moorei* in the presence of RGD peptides abrogated the *S. moorei*-induced integrin α2/β1-PI3K-AKT-mTOR-C-myc signalling cascade and PCNA protein expression (Figure 5C-D and S3B-C). RGD peptides also blocked the increase in clone formation of DLD-1 and HT-29 cells induced by* S. moorei* while RGDS alone has no significant effect on clone formation. (Figure S3D-E). siRNA-mediated silencing of integrin α2/β1 in CRC cells also showed similar effects following incubation with *S. moorei*, integrin α2, integrin β1, p-FAK, p-PI3K, p-AKT, p-mTOR, C-myc, and PCNA protein levels were also downregulated in CRC cells exposed to *S. moorei* following transfection with siRNA targeting integrin α2/β1 (Figure 5E-F and S3F-G). The above findings corroborated that *S. moorei* induced integrin α2/β1-PI3K-AKT-mTOR-C-myc signaling pathway in CRC cells.

### *S. moorei* activated the integrin α2/β1 signaling cascade and blockade of integrin α2/β1 inhibited tumorigenic and signaling activities of *S. moorei in vivo*

**Figure 6:**
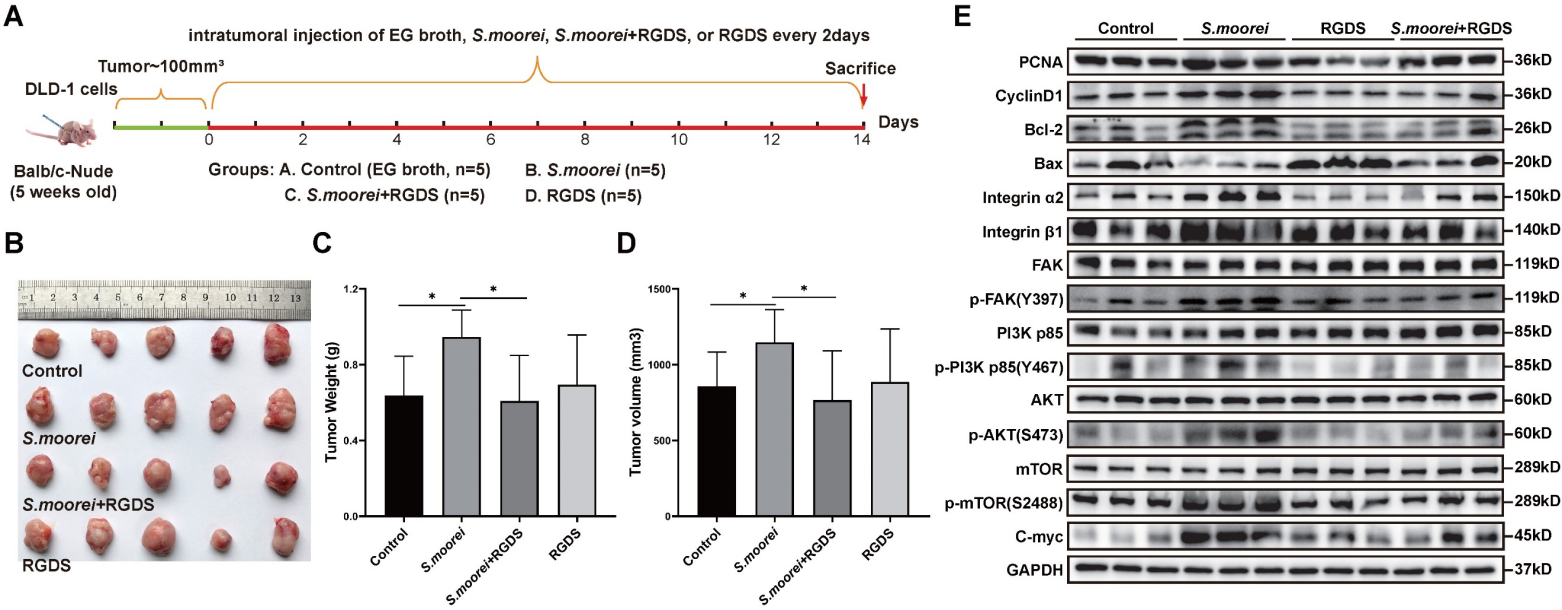
***S. moorei* activates the integrin α2/β1 signaling cascade and blockade of integrin α2/β1 inhibited tumorigenic and signaling activities of *S. moorei* in DLD-1 xenograft tumor model.** (A) Schematic diagram showing the experimental design and timeline of DLD-1 xenograft tumor model. (B) The images of DLD-1 xenograft tumor under different treatment. (C) The tumor weight under different treatment. (D) The tumor volume under different treatment. (E) Protein expression of proliferation/apoptosis-related genes and integrin α2/β1-FAK-PI3K-AKT-mTOR-C-myc signaling pathway in tumors of DLD-1 xenograft tumor model under different treatment. RGDS, RGD peptides.

To investigate the *in vivo* effect of *S. moorei* in altering cell signaling pathways, qRT-PCR assay was conducted to analyze gene expression levels in colonic tumors of *Apc^Min/+^* mice receiving *S. moorei* or vehicle treatment. Expression levels of Pcna, Ccnd1(CyclinD1), Bcl2, Itga2, Itgb1, Fak, Pik3r1, Akt1, Mtor and Myc were remarkably upregulated and Bax was downregulated in colonic tumors of mice treated with *S. moorei* (Figure S4A). Moreover, the expression of PCNA, CyclinD1, Bcl-2, Integrin α2/β1, p-FAK, p-PI3K (p85), p-AKT, p-mTOR, C-myc proteins was also significantly increased in the tumors of *Apc^Min/+^* mice treated with *S. moorei* (Figure S4B). Then, the DLD-1 xenograft tumor model was used to investigate the role of *S. moorei* in promoting colorectal carcinogenesis and the effect of integrin α2/β1 signaling blockade on the tumorigenic activities of *S. moorei*. Mice with subcutaneous colorectal cancer were intratumorally injected with EG broth, *S. moorei*, RGD peptides, and *S. moorei* plus RGD peptides and the tumor tissue was harvested 2 weeks later (Figure 6A). The introduction of *S. moorei* into the subcutaneous xenografts in mice increased tumor weight and volume but RGD peptides treatment reversed the effect of *S. moorei* on the increase in tumor weight and volume (Figure 6B-D). The use of EG both or RGD peptides alone had no effect on tumor growth. In addition, protein expression of PCNA, CyclinD1, Bcl-2, Integrin α2/β1, p-FAK, p-PI3K (p85), p-AKT, p-mTOR, C-myc proteins were also significantly increased in the DLD-1 xenograft tumor treated with *S. moorei* and were reversed by RGD peptides in *S. moorei*-treated DLD-1 xenograft tumor (Figure 6E). These findings were consistent with *in vitro* results, indicating that *S. moorei* contributed to the activation of integrin α2/β1-PI3K-AKT-mTOR-C-myc signaling cascade.

### The *S. moorei* cellwall protein Cna B-type domain-containing protein binds to integrin α2/β1 on CRC cells

**Figure 7:**
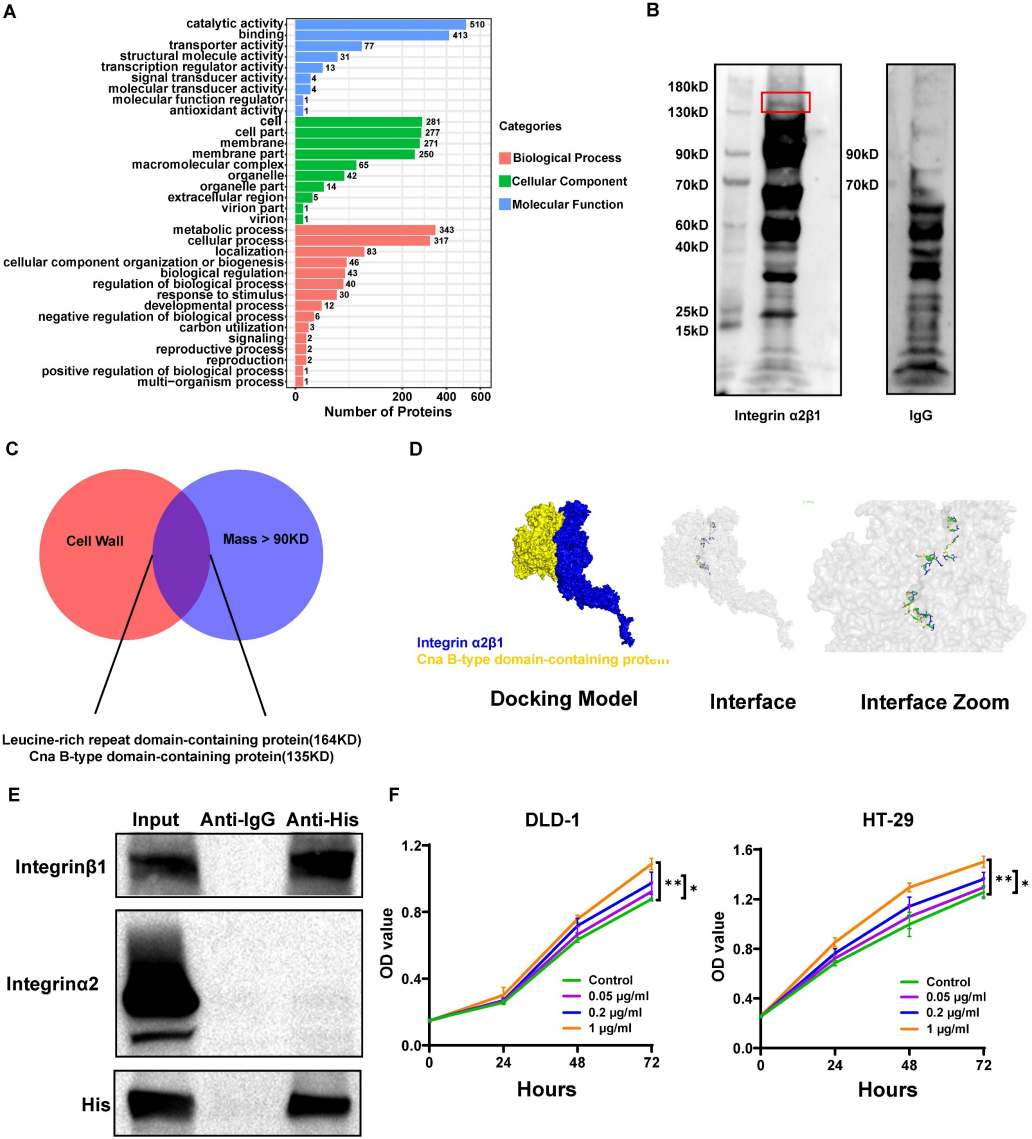
**The *S. moorei* cellwall protein Cna B-type domain-containing protein binds to integrin α2/β1 on CRC cells.** (A) Gene ontology for protein of *S. moorei*. (B) Far western blotting for protein of *S. moorei* interacted with integrinα2/β1. (C) Venn diagram for protein identified location on cell wall and mass over than 90KD. (D) Surface diagram of the docking model and their interfacing residues between Cna B-type domain-containing protein and integrinα2/β1. (E) Co-IP analysis demonstrating the interaction between the Cna B-type domain-containing protein (with His-tag) and integrin α2/β1, confirming the binding of the B-type domain-containing protein to integrin β1. (F) The effect of different concentrations of recombinant Cna B-type domain-containing protein on the proliferation of DLD-1 and HT-29 cells.

**Figure 8:**
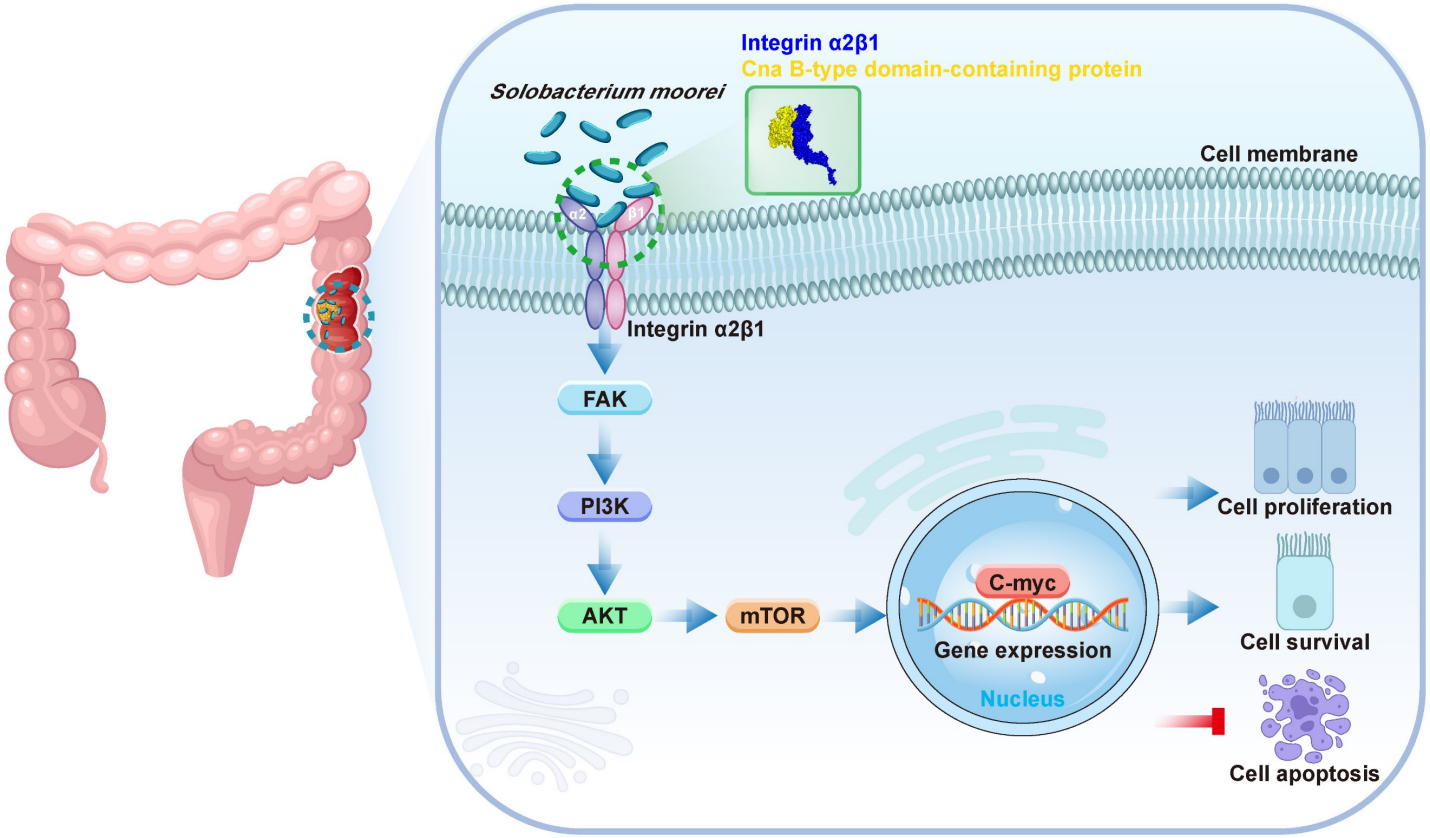
Schematic model depicting the proposed mechanism of *S. moorei*-mediated progression of colorectal cancer.

Since *S. moorei* attached preferentially to CRC cells and activates the integrin α2/β1- signaling pathway, we next to identify the surface proteins capable binding to the integrin α2/β1 of CRC cells. We first identified 805 proteins of *S. moorei* through mass spectrometry and clarified their localization (Table S10). Among the 805 proteins mentioned above, 10 proteins are located on the cellwall. Figure 7A shows the GO pathway enriched with *S. moorei* proteins. Using the far-western method, we identified the corresponding bands of protein from *S. moorei* that interacted with human integrin α2/β1 heterodimer protein. The results showed that compared with the IgG control group, the molecular mass of the *S. moorei* protein significantly bound to integrin α2/β1 was greater than 90 kDa (Figure 7B). Leucine-rich repeat domain-containing protein (164 kDa) and Cna B-type domain-containing protein (135 kDa) were *S. moorei* cellwall proteins which have a molecular mass greater than 90 kDa (Figure 7C). Considering that the protein band is closer to the molecular mass of 130 kDa, we speculate that Cna B-type domain-containing protein is the *S. moorei* cellwall protein binding to integrin α2/β1. To validate the binding of *S. moorei* Cna B-type domain-containing protein to integrin α2/β1 heterodimer protein, we performed rigid protein-protein docking between integrin α2/β1 and Cna B-type domain-containing protein. As shown in Figure 7D, integrin α2/β1 and Cna B-type domain-containing protein conjugated together through hydrogen bonds which formed by amino acid residue sites, revealing they formed a stable docking model (Supplementary Table S11). We subsequently constructed a plasmid encoding the Cna B-type domain-containing protein with a His tag and transfected it into *E. coli* BL21(DE3) cells for protein expression (Figures S5A-S5B). Results from the Co-IP experiments indicated that the Cna B-type domain-containing protein binds to integrin β1 in CRC cells (Figure 7E). Additionally, we assessed the impact of the recombinant Cna B-type domain-containing protein on CRC cell proliferation. The findings revealed that at concentrations of 0.2 and 1 μg/ml, the Cna B-type domain-containing protein significantly enhanced the proliferation and colony formation of DLD-1 and HT-29 cells (Figure 7F and S5C) while markedly inhibiting their apoptosis (Figure S5D). These results suggest that the Cna B-type domain-containing protein may play a crucial role in the oncogenic activity of *S. moorei*. Collectively, our findings validate the underlying role of the integrin α2/β1-PI3K-AKT-mTOR-C-myc signaling cascade in *S. moorei*-mediated colorectal tumorigenesis (Figure 8).

## Discussion

Microbial dysbiosis has been increasingly suggested to facilitate the progression of CRC^35^. Metagenomic profiling was conducted on fecal and mucosal specimens collected from CRC patients, which suggested that *S. moorei* was one of the oncogenic bacteria that is highly enriched in CRC^22^, ^23^. The present study confirmed that *S. moorei* was enriched in CRC and was also significantly correlated with tumor staging. The present study confirmed that *S. moorei* was enriched in CRC by qRT-PCR analysis. *S. moorei* was enriched in fecal specimens of CRC patients in comparison to healthy subjects. Moreover, *S. moorei* was highly enriched in tumor tissues of CRC patients. Following microbiota depletion, *S. moorei* treatment dramatically accelerated the progression of CRC compared to the *Apc^Min/+^* or AOM/DSS-treated mice exposed to *E. coli* strain MG1655. In addition, *S. moorei* promoted the growth of CRC cells. Collectively, our data suggested that *S. moorei* infection was linked to CRC and might be a candidate driver bacterium responsible for the progression of CRC. The mechanism of *S. moorei*-mediated progression of CRC is still unclear. The present work elucidated a mechanism by which *S. moorei* promoted cell proliferation in CRC. *S. moorei* particularly accumulated in the tumor tissue, indicating its possible interaction with CRC cells. Similar to *in vivo* findings, *S. moorei* showed attachment to CRC cells and colonic epithelial cells *in vitro*; however, the attachment efficiency of *S. moorei* was much higher for CRC cells compared to colonic epithelial cells. Notably, the attachment and colonization of cancer-promoting bacteria created a conducive environment for cancer progression, which was observed during* F. nucleatum, B. fragilis, P. anaerobius*, and *P. gingivalis* infections^12^, ^36^-^38^. We detected an altered gene expression profile in CRC cells after *S. moorei* treatment using transcriptome sequencing. Treatment with *S. moorei* induced the expression of ITGA2, ITGB1, and MYC genes in CRC cells. Gene enrichment analysis showed significant changes in the expression of genes involved in the PI3K-AKT pathway.

Integrins are the surface adhesion receptors mediating interactions between neighboring cells and the extracellular matrix^39^. Following binding to their ligands, integrins activate cell signaling pathways by recruiting and activating signaling proteins, including FAK^40^. FAK is responsible for the phosphorylation of different adaptor proteins, like PI3K, and subsequently activating the PI3K-AKT pathway^41^. Activated FAK-PI3K-AKT pathway accelerates cell motility, cell survival, and cell cycle progression^42^. FAK-PI3K-AKT pathway has been found to be activated in different cancer types and induces the proliferation and migration of tumor cells^43^, ^44^. It was reported that integrin α2/β1 complex was overexpressed in CRC cells compared to colonic epithelial cells^12^, which might contribute to increased *S. moorei* attachment to tumor tissues. Additionally, *S. moorei* stimulated the expression of integrin α2/β1 levels in CRC cells, promoting cell adhesion. Likewise, pathogenic bacteria are shown to bind to integrins of CRC cells during pathogenesis. In tumor tissues from *S. moorei*-exposed *Apc^Min/+^* mice and cultured CRC cells, integrin α2/β1 complex induced FAK phosphorylation which subsequently activated PI3K-AKT-mTOR-C-myc oncogenic signaling cascade. Indeed, *S. moorei* promoted cell growth in the *Apc^Min/+^* and AOM/DSS-treated mice models and two CRC cell lines *in vitro*. RGD peptides or siRNA targeting integrin α2/β1 abolished *S. moorei*-mediated cell growth and the activation of the PI3K-AKT pathway, which suggested that the binding of bacteria to integrin α2/β1 was necessary for the tumor-promoting activity of *S. moorei*. In addition, we have preliminarily explored the ligand on the surface of *S. moorei* that binds to integrin α2/β1 on CRC cells and speculated that *S. moorei* cellwall protein Cna B-type domain-containing protein might bind to integrin α2/β1. According to the different binding characteristics of integrins, they can be divided into four types: leukocyte adhesion integrin, RGD binding integrin, collagen binding integrin, and laminin binding integrin. Integrin α2/β1 is one of collagen binding integrins which expressed on epithelial cells^45^. Through the docking model (Figure 7D), we have found Cna B-type domain-containing protein conjugate with I domain (AA:140-359) of integrinα2β1 which is also the key domain in binding of collagen^46^, ^47^. Not only that, we further discovered Cna B-type domain-containing protein conjugated to integrin β1 and promoted CRC cells growth which determined its role in cancer progression (Figure 7E-F). In our research, we found RGD peptides, which had been reported regulating conjugation of collagen and integrin^48^, impended integrin downstream signal provoked by *S. moorei*. Thus, we speculated Cna B-type domain-containing protein might play roles like collagen which activated cancer cell proliferation and migration.

## Conclusions

In summary, we found that *S. moorei* was enriched in fecal samples from CRC patients compared to healthy subjects and was also enriched in CRC tissues from *Apc^Min/+^* mice compared to matched non-tumor tissues. *S. moorei* abundance was significantly correlated with tumor staging. *S. moorei* cellwall protein Cna B-type domain-containing protein interacted with integrin α2/β1, which was overexpressed on CRC cells, leading to the activation of PI3K-AKT-mTOR-C-myc pathway via phospho-FAK; thus, promoting cell growth and tumor progression. This work explored the effect of *S. moorei* on the progression of CRC and identified a potential molecular mechanism by which *S. moorei* promoted CRC progression. This study suggests that *S. moorei* might be a new potential target for CRC prevention, diagnosis, and treatment.

## Supplementary Material

Supplementary figures and tables 1-4, 11.

Supplementary table 5.

Supplementary table 6.

Supplementary table 7.

Supplementary table 8.

Supplementary table 9.

Supplementary table 10.
