# Supplementary material for: Solobacterium moorei promotes tumor progression via the Integrin α2/β1-PI3K-AKT-mTOR-C-myc signaling pathway in colorectal cancer
Source: Int J Biol Sci. 2025 Jan 27;21(4):1497–512. doi: 10.7150/ijbs.102742 (PMC11844286; doi:10.7150/ijbs.102742)
Supplement: Supplementary file 1 — Supplementary figures and tables 1-4, 11. [file ijbsv21p1497s1.pdf]

Solobacterium moorei promotes tumor progression via the Integrin α2/β1-PI3K-AKT-mTOR-C-myc signaling pathway in colorectal cancer

Yan Chen, Ying Qin, Tingting Fan, Cheng Qiu, Yijie Zhang, Mengmeng Dai, Yaoyao Zhou, Qinsheng Sun, Yuan Guo, Yue Hao, Yuyang Jiang

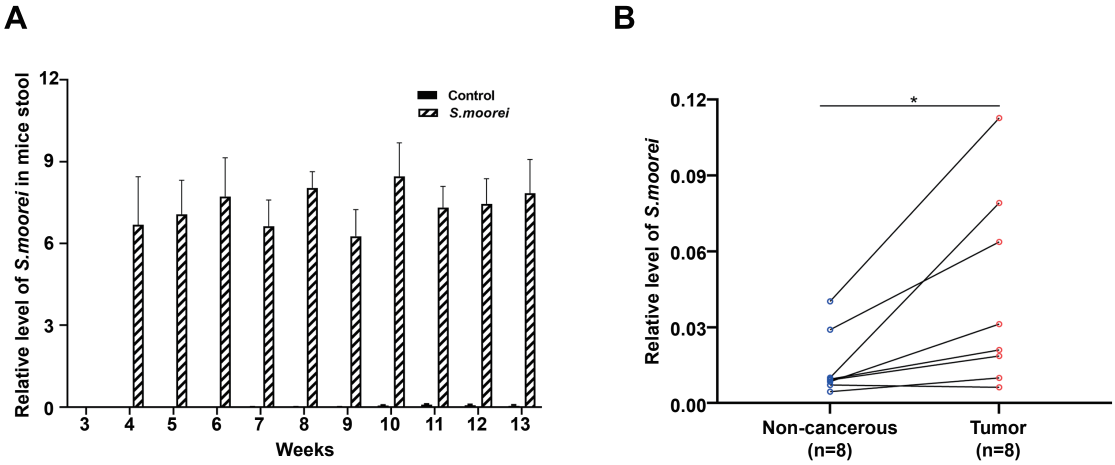

Figure S1. *S. moorei* abundance in feces and tumor tissues of *Apc*$^{Min/+}$ mice. (A) The level of *S. moorei* in stool samples of *Apc*$^{Min/+}$ mice during *S. moorei* feeding period by qRT-PCR. (B) *S. moorei* was enriched in colonic tumors compared with adjacent normal tissues in *Apc*$^{Min/+}$ mice treated with *S. moorei*.

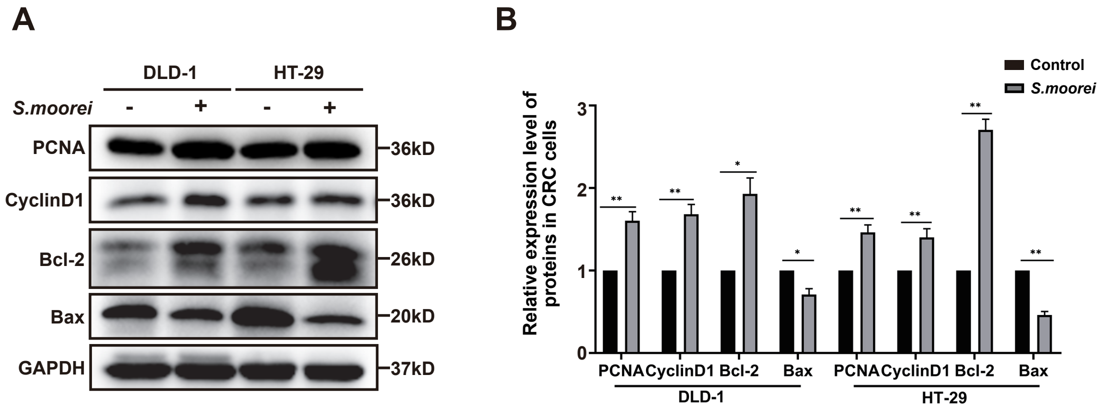

Figure S2. ***S. moorei*** **induced the changes of proliferation/apoptosis-related protein expression in CRC cells.** (A) Protein expression of proliferation/apoptosis- related protein expression in CRC cells co-cultured with *S. moorei*. (B) Quantification of protein expression for all blots showed in (A).

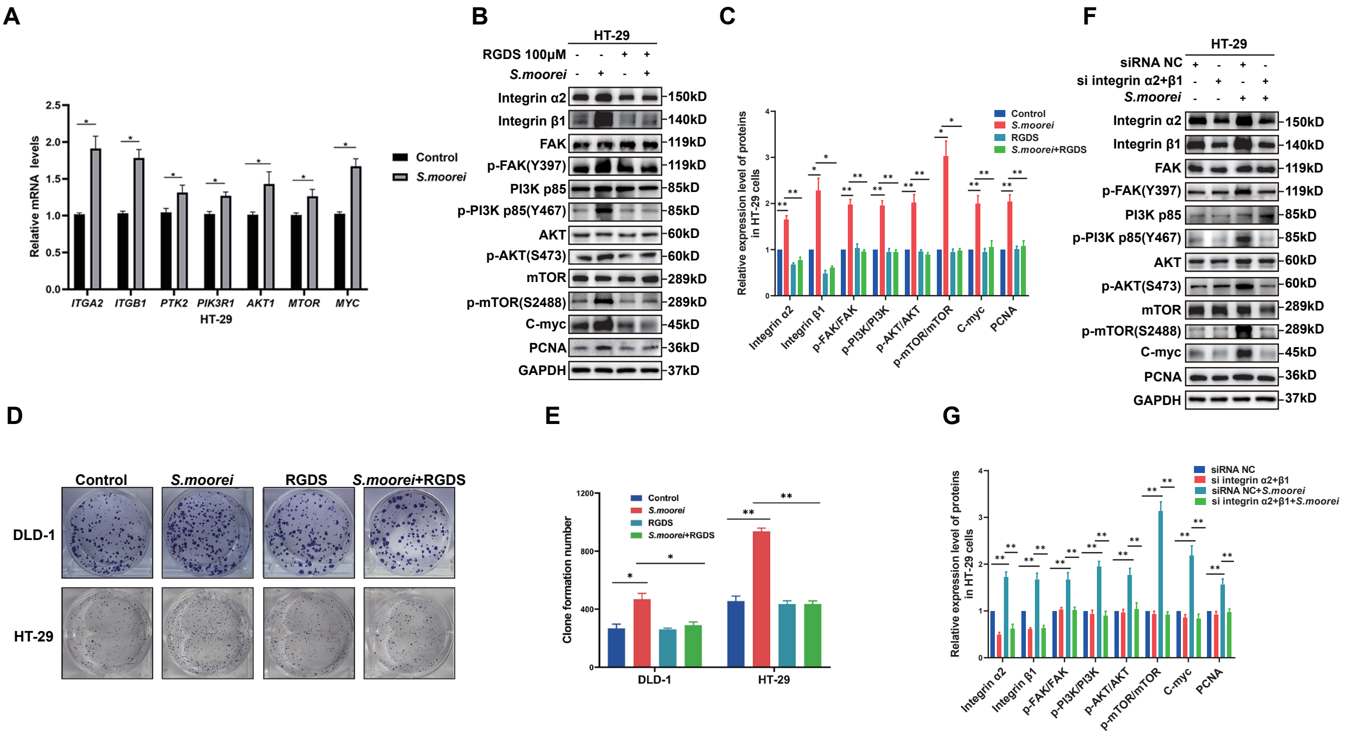

Figure S3. *S. moorei* mediates integrin α2/β1 signaling to activate the FAK-PI3K-AKT-mTOR-C-myc pathway to promote cell proliferation in HT-29 cells. (A) Gene expression of integrin α2/β1-FAK-PI3K-AKT-mTOR-C-myc signaling pathway in HT-29 cells co-cultured with *S. moorei*. (B) Protein expression of integrin α2/β1-FAK-PI3K-AKT-mTOR-C-myc signaling pathway triggered by *S. moorei* in HT-29 cells. The effects of *S. moorei* were abolished by the integrin inhibitor RGD peptides (100 μM). (C) Quantification of protein expression for all blots showed in (B). (D) RGD peptides blocked the increase in clone formation of DLD-1 and HT-29 cells induced by *S. moorei*. (E) The colony formation number of DLD-1 and HT-29 treated S. moorei and RGD peptides. (F) Integrin α2/β1 knockdown abolished the effect of S. moorei on the integrin α2/β1-FAK-PI3K-AKT-mTOR-C-myc pathway in HT-29 cells. (G) Quantification of protein expression for all blots showed in (F).

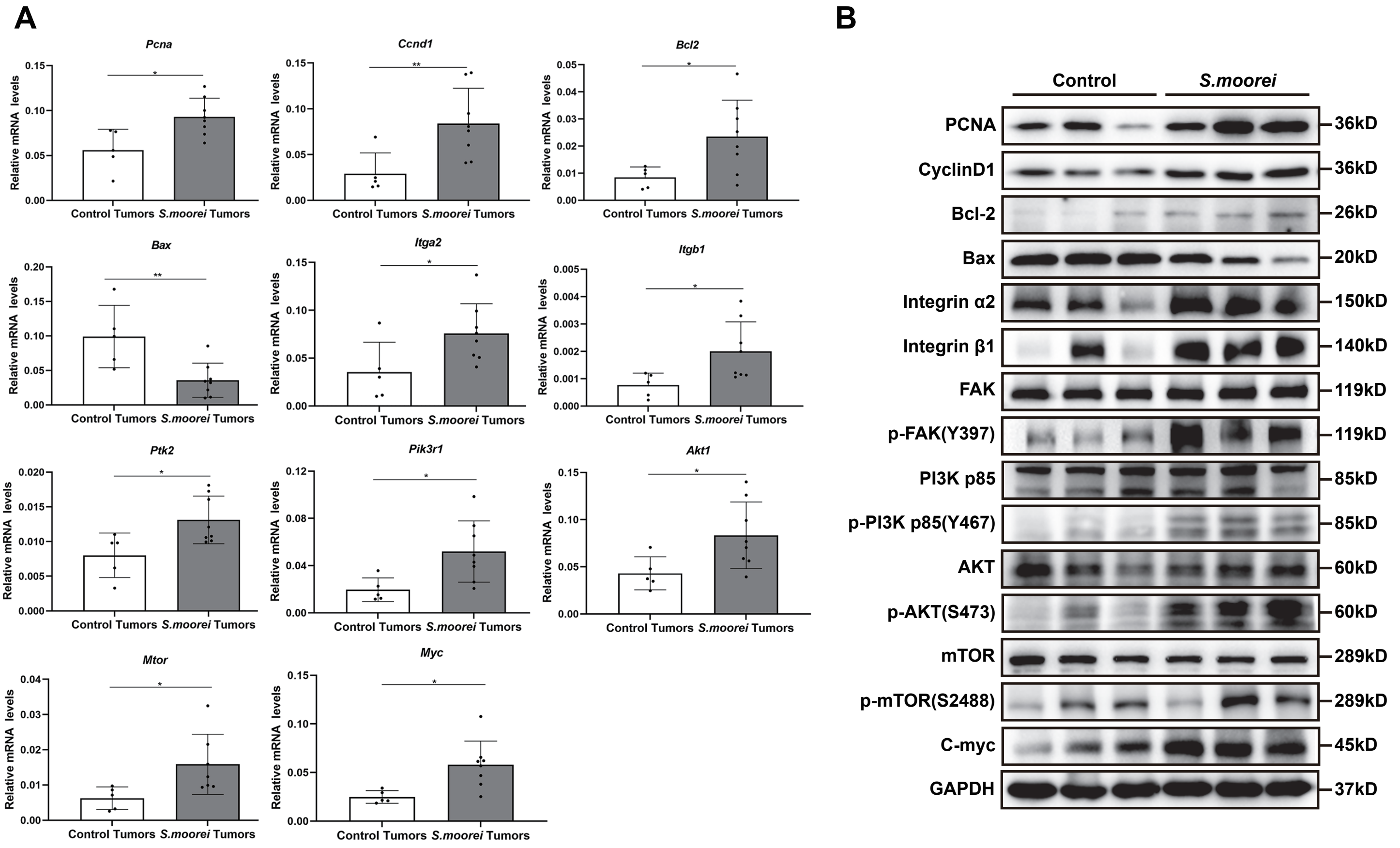

Figure S4. *S. moorei* activates the integrin α2/β1-PI3K-AKT-mTOR-C-myc signaling cascade in *Apc*$^{Min/+}$ mice. (A) Gene expression of proliferation/apoptosis-related genes and integrin α2/β1-FAK-PI3K-AKT-mTOR-C-myc signaling pathway in tumors of *Apc*$^{Min/+}$ mice treated with *S. moorei* or vehicle. (B) Protein expression of proliferation/apoptosis-related genes and integrin α2/β1-FAK-PI3K-AKT-mTOR-C-myc signaling pathway in tumors of *Apc*$^{Min/+}$ mice treated with *S. moorei* or vehicle.

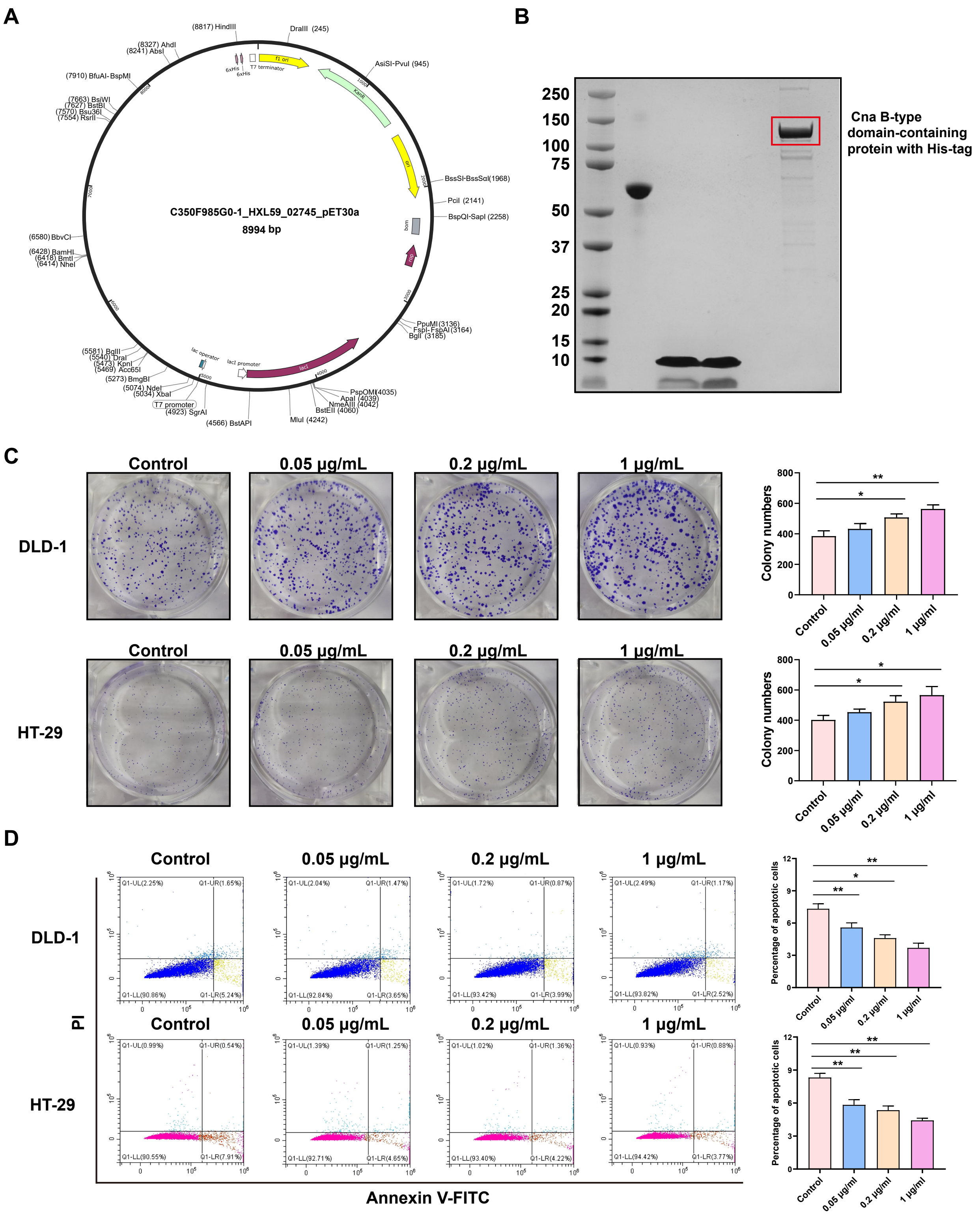

Figure S5. The oncogenic effect of the *S. moorei* cellwall protein Cna B-type domain-containing protein in CRC. (A) Sequence diagram of plasmid containing Cna B-type domain-containing protein with His tag. (B) SDS-PAGE used to detect the expression of the recombinant Cna B-type domain-containing protein with His-tag. (C) The effect of different concentrations of recombinant Cna B-type domain-containing protein on the colony formation of DLD-1 and HT-29 cells. (D) The effect of different concentrations of recombinant Cna B-type domain-containing protein on the apoptosis of DLD-1 and HT-29 cells.

Table S1. The clinicopathological parameters of 89 CRC patients

Parameters	Number of patients (%）
**Gender**	
male	49(55.1%)
female	40(44.9%)
**Age**	
≤50	23(25.8%)
＞50	66(74.2%)
**TNM stage**	
I+II	47(52.8%)
III+IV	42(47.2%)
**Lymph nodes metastasis**	
N0	47(52.8%)
N1+N2	42(47.2%)
**Distant metastasis**	
M0	80(89.9%)
M1	9(10.1%)

N0: No regional lymph node metastasis; N1: Metastasis in 1–3 regional lymph nodes; N2: Metastasis in 4 or more regional lymph nodes. M0: No distant metastasis; M1: Distant metastasis.

Table S2. Primer sequences used for qRT-PCR

Gene	Species	Sequence (5'-3')	References
S. moorei-F	bacteria	CTCAACCCAATCCAGCCACT	[27]
S. moorei-R		TATTGGCTCCCCACGGTTTC	
Eubacteria 16S-F	bacteria	GGTGAATACGTTCCCGG	[11]
Eubacteria 16S-R		TACGGCTACCTTGT TACGACTT	
ITGA2-F	human	GGGAATCAGTATTACACAACGGG	[12]
ITGA2-R		CCACAACATCTATGAGGGAAGGG	
ITGB1-F	human	GTAACCAACCGTAGCAAAGGA	[12]
ITGB1-R		TCCCCTGATCTTAATCGCAAAAC	
PTK2-F	human	TACAACGAGGGTGTCAAGCC	[28]
PTK2-R		GCCCGTCACATTCTCGTACA	
PIK3R1-F	human	TGGACGGCGAAGTAAAGCATT	[12]
PIK3R1-R		AGTGTGACATTGAGGGAGTCG	
AKT1-F	human	AGCGACGTGGCTATTGTGAAG	[12]
AKT1-R		GCCATCATTCTTGAGGAGGAAGT	
MTOR-F	human	TCCGAGAGATGAGTCAAGAGG	[29]
MTOR-R		CACCTTCCACTCCTATGAGGC	
MYC-F	human	AAGAGGGTCAAGTTGGACAGTTGC	[30]
MYC-R		TTTCGGTTGTTGCTGATCTGTCT	
GAPDH-F	human	GCACCGTCAAGGCTGAGAAC	[31]
GAPDH-R		TGGTGAAGACGCCAGTGGA	
Pcna-F	mouse	CTTACTCTGCGCTCCGAAGG	Designed in this
Pcna-R		TTGGACATGCTGGTGAGGTT	study
Ccnd1-F	mouse	GCGTACCCTGACACCAATCT	Designed in this
Ccnd1-R		TTTTCCGCATGGATGGCACA	study
Bcl2-F	mouse	AGCCTGAGAGCAACCCAATG	Designed in this
Bcl2-R		TGACCCCACCGAACTCAAAG	study
Bax-F	mouse	TGTGCACTAAAGTGCCCGAG	Designed in this
Bax-R		ATGTGGGGGTCCCGAAGTAG	study
Itga2-F	mouse	TGTCTGGCGTATAATGTTGGC	[12]
Itga2-R		CTTGTGGGTTCGTAAGCTGCT	
Itgb1-F	mouse	ATGCCAAATCTTGCGGAGAAT	[12]
Itgb1-R		TTTGCTGCGATTGGTGACATT	
Ptk2-F	mouse	CGGACACATGCAGTCTCTGT	[28]
Ptk2-R		CGAGGGCATGGTGTATGTGT	
Pik3r1-F	mouse	ACACCACGGTTTGGACTATGG	[12]
Pik3r1-R		GGCTACAGTAGTGGGCTTGG	
Akt1-F	mouse	ATGAACGACGTAGCCATTGTG	[12]
Akt1-R		TTGTAGCCAATAAAGGTGCCAT	
Mtor-F	mouse	CCGTTATGTCGATGGTCGGA	Designed in this
Mtor-R		TTGCCATCCAGACCCGTAAC	study

Myc-F	mouse	GGATTTCCTTTGGGCGTTGG	Designed in this study
Myc-R		GCTGTACGGAGTCGTAGTCG	
Gapdh-F	mouse	GGCAAATTCAACGGCACAGT	[28]
Gapdh-R		AGATGGTGATGGGCTTCCC	

Table S3. Antibodies used in this study

Antibody	Catalog No.	Company	Dilution
PCNA	#2586S	CST	1:1000
CyclinD1	#55506S	CST	1:1000
Bcl-2	#15071T	CST	1:1000
Bax	#5023S	CST	1:1000
Integrin β1	ab52971	Abcam	1:10000
Integrin α2	ab133557	Abcam	1:10000
FAK	ab40794	Abcam	1:1000
Phospho-FAK (Y397)	ab81298	Abcam	1:1000
PI3Kp85α	ab86714	Abcam	1:1000
Phospho-PI3Kp85/p55 (Y467/Y199)	ab278545	Abcam	1:1000
Akt	#9272s	CST	1:5000
Phospho-Akt (S473)	#9271s	CST	1:1000
mTOR	ab32028	Abcam	1:1000
Phospho-mTOR (S2448)	ab109268	Abcam	1:1000
C-myc	ab32072	Abcam	1:1000
GAPDH	ab8245	Abcam	1:10000

Table S4. siRNA and negative control sequences

**Gene**	**Sense (5'-3')**	**Antisense (5' -3')**
ITGB1	GCACCAGCCCAUUUAGCUA	UAGCUAAWGGGCUGGUGC
ITGA2	GUGGUUGUGUGUGAUGAAU	AUUCAUCACACACAACCAC
negative control	UUCUCCGAACGUGUCACGU	ACGUGACACGUUCGGAGAA

Table S11. Hydrogen Bond and Salt Bridge between Integrinα2/β1 and Cna B-type domain-containing protein

Interaction	Integrin	Distance	Cna B-type domain-containing protein
Hydrogen Bond	A: THR 89[N]	3.34	D:PRO 881[O]
	A: LYS 94[NZ]	3.58	D: GLY 534[O]
	A: THR 99[OG1]	2.39	D: GLU 833[OE2]
	A: ARG 612[NH2]	3.45	D:ASP 958[OD2]
	A: GLN 690[NE2]	3.34	D: ILE 953[O]
	A: SER 768[N]	3.3	D: SER 951[OG]
	A: ARG 612[O]	2.08	D: THR 473[OG1]
	A: MET 77[SD]	2.11	D: LYS 537[N]
	A: ASN 96[O]	2.24	D: LYS 537[NZ]
	A: THR 111[OG1]	3.72	D: ASN 582[ND2]
	A: GLY 140[O]	2.75	D: GLN 583[NE2]
	A:ASP 203[OD2]	2.9	D: THR 593[OG1]
	A:ASP 203[OD1]	2.63	D: THR 593[OG1]
	A: THR 207[OG1]	2.17	D: SER 595[N]
	A: THR 207[O]	3.51	D: SER 595[OG]
	A: SER 88[OG]	3.78	D: ASP 883[N]
	A: GLU 36[OE1]	3.61	D: ASN 885[ND2]
	A:PRO 765[O]	3.65	D: MET 946[N]
	B: THR 178[OG1]	3.45	D: ASN 582[ND2]
Salt Bridge	A: ARG 612[NH1]	3.87	D: ASP 958[OD2]
	A: ARG 612[NH2]	3.45	D: ASP 958[OD2]
	A: GLU 36[OE1]	3.87	D: LYS 957[NZ]
